# The vision of Dutch paediatric care until the year 2030: prevention and collaboration as key ingredients

**DOI:** 10.1093/eurpub/ckac129.601

**Published:** 2022-10-25

**Authors:** D Jansen, K Illy

**Affiliations:** 1 Department of General Practice and Elderly Care Medicine, University Medical Center Groningen, Groningen, Netherlands; 2 Dutch Paediatric Society, Utrecht, Netherlands

## Abstract

There are many societal developments in The Netherlands that have or will have an impact on Dutch paediatric care. These developments both reveal potential risks in paediatric care and require future improvement of paediatric practices to achieve the best possible outcomes for Dutch children. To realise this, the Dutch Paediatric Society decided in their renewed vision to have a closer focus on prevention and on building partnership with public health, by implementing a building block ‘Interprofessional Collaboration between Paediatricians and Other Health Care Providers’. This building block formulates the wish of the Dutch Paediatric Society to work together in networks with other domains of care, such as public health. Preferably, it will be a flexible network in which the disciplines involved align the needs of the specific child. In order to develop and participate in such a network, pediatricians and public health professionals actively have to invest in connecting, establishing, and further developing professional networks (including patient societies). A first precondition for the development and successful functioning of such a network is the implementation and use of a joint electronic medical record in which all diagnostics, treatment plans and positive health aspects of the child are collected, and which is accessible to all professionals involved. A patient record which guarantees a barrier-free exchange of medical and non-medical information between paediatricians and public health professionals within the framework of the current privacy legislation. A second precondition regards an improvement of the paediatric training curriculum in which the paediatrician of the future will gain knowledge of positive health and integrative medicine. The future paediatrician must be trained to function in networks and to make connections. In this presentation we present an update of the implementation of this building block: what is already realised and how.

